# Chlorophyll fluorescence analysis revealed essential roles of FtsH11 protease in regulation of the adaptive responses of photosynthetic systems to high temperature

**DOI:** 10.1186/s12870-018-1228-2

**Published:** 2018-01-10

**Authors:** Junping Chen, John J. Burke, Zhanguo Xin

**Affiliations:** Plant Stress and Germplasm Development Unit, USDA-ARS, 3810 4th Street, Lubbock, TX 79415 USA

**Keywords:** Heat stress, Thermotolerance, Photosynthesis; Thermostability, FtsH11, Chlorophyll fluorescence, High temperature

## Abstract

**Background:**

Photosynthetic systems are known to be sensitive to high temperature stress. To maintain a relatively “normal” level of photosynthetic activities, plants employ a variety of adaptive mechanisms in response to environmental temperature fluctuations. Previously, we reported that the chloroplast-targeted AtFtsH11 protease played an essential role for Arabidopsis plants to survive at high temperatures and to maintain normal photosynthetic efficiency at moderately elevated temperature. To investigate the factors contributing to the photosynthetic changes in *FtsH11* mutant, we performed detailed chlorophyll fluorescence analyses of dark-adapted mutant plants and compared them to Col-0 WT plants under normal, two moderate high temperatures, and a high light conditions.

**Results:**

We found that mutation of *FtsH11* gene caused significant decreases in photosynthetic efficiency of photosystems when environmental temperature raised above optimal. Under moderately high temperatures, the *FtsH11* mutant showed significant 1) decreases in electron transfer rates of photosystem II (PSII) and photosystem I (PSI), 2) decreases in photosynthetic capabilities of PSII and PSI, 3) increases in non-photochemical quenching, and a host of other chlorophyll fluorescence parameter changes. We also found that the degrees of these negative changes for utilizing the absorbed light energy for photosynthesis in *FtsH11* mutant were correlated with the level and duration of the heat treatments. For plants grown under normal temperature and subjected to the high light treatment, no significant difference in chlorophyll fluorescence parameters was found between the *FtsH11* mutant and Col-0 WT plants.

**Conclusions:**

The results of this study show that AtFtsH11 is essential for normal photosynthetic function under moderately elevated temperatures. The results also suggest that the network mediated by AtFtsH11 protease plays critical roles for maintaining the thermostability and possibly structural integrity of both photosystems under elevated temperatures. Elucidating the underlying mechanisms of FtsH11 protease in photosystems may lead to improvement of photosynthetic efficiency under heat stress conditions, hence, plant productivity.

**Electronic supplementary material:**

The online version of this article (10.1186/s12870-018-1228-2) contains supplementary material, which is available to authorized users.

## Background

Photosynthesis is the primary source of raw materials (carbohydrates, amino acids, fatty acids, etc.) for all forms of agricultural products. In nature, plants are subject to daily and seasonal temperature fluctuations in their growing environments. Therefore, photosynthetic systems are under frequent influence of temperature changes. Studies have shown that chloroplast membranes, especially thylakoid membranes and associated photosynthetic apparatus, are extremely sensitive to high temperature (heat stress) and are thought to be the primary targets of heat stress [1–4]. High temperature extremes have profound negative impacts on essentially all aspects of photosynthetic activities, from changes in enzyme activities and photochemical reactions, damages to photosystem II proteins, alternations in membrane fluidity and photosystem protein complexes, to the irreversible destruction of the entire PSII [2–7]. As the trend of global temperature increases [8, 9], the negative effects of heat stress on photosynthesis, hence on world agriculture production and food security are expected to exacerbate [10–13]. Understanding the basic mechanisms by which photosynthetic systems in plants respond and adapt to temperature upshifts in their environment is a prerequisite for any attempt to enhance or even maintain efficient photosynthesis under heat stress conditions. However, comparing to the studies on functions of FtsH proteases under light stress [14, 15], very little is known on how plants sense the temperature changes and regulate those essential responses to maintain relatively “normal” photosynthetic activities under moderate high temperatures.

Previously, we reported that AtFtsH11, a homolog of a filamentous temperature sensitive H protease (FtsH), plays an essential role in thermotolerance of Arabidopsis plants [16]. Mutations of AtFtsH11 cause reduction in photosynthetic efficiency under moderately elevated temperature of 30 °C. The FtsH proteases are a class of membrane-bound, ATP-dependent zinc-metalloprotease found in all eukaryotic organisms examined so far [17, 18]. In prokaryotic and yeast systems, FtsH proteases are essential to microbial survival at high temperature and are also involved in protection against other environmental stresses [19–21]. These proteases function as molecular chaperones and help organisms to maintain cell homeostasis under stress conditions [22–24]. Plant genomes contain multiple FtsH homolog genes, with most of them predicted to be either chloroplast or mitochondria targeted [15, 25, 26]. In Arabidopsis, major chloroplast targeted FtsH proteases (FtsH1/5, FtsH2/8, FtsH6 and FtsH7/9) have been shown to function in alleviating and repairing high light induced photodamages to photosystems (PS), especially in the turnover of photodamaged D1 protein in PSII [14, 25, 27–34]. The FtsH11 is the first plant FtsH protease reported to be involved in thermotolerance in plants. Recently, a role of FtsH6 in the regulation of HSP21 level has also be proposed [35].

Chlorophyll (Chl) fluorescence measurements have been widely used to examine the impacts of a variety of abiotic stresses on photosynthetic systems in plants [36–40]. The effects of high temperatures on photosynthetic activity and apparatus have been evaluated by both slow and fast Chl fluorescence kinetic measurements in many plant species, such as Arabidopsis, wheat and beans [41–44]. High temperature induced reduction in the efficiency of PSII photochemistry and PS1 functions have been revealed using the Chl fluorescence measurements [39, 41, 44]. Moreover, with the improved technology, Chl fluorescence has become a powerful tool for detailed analysis of functional and structural changes of PS under a variety environmental stresses and in characterization of heat stress tolerance of plant varieties [37, 38, 40, 42]. In Arabidopsis *FtsH11* mutants, the decrease in thermotolerance appears to be associated with decreased photosynthetic efficiency (quantum yield) above 30 °C [16]. However, which specific photosynthetic factors contribute to such decreases, and exactly how mutation of FtsH11 altered their normal functions are still unknown and need to be investigated. In this study, we applied the Chl florescence approach to examine the effects of *FtsH11* mutation on a range of photosynthetic parameters under normal and moderately high temperature conditions in Col-0 and *Ftsh11* mutant plants. We identified the major Chl fluorescence parameters that were affected by Ftsh11 mutations and how their changes contributed to the detected decreases in PSII efficiency in *FtsH11* mutant under moderate high temperatures. The study also revealed that mutations in *FtsH11* gene had much broader negative impact on photosynthetic systems in plants.

## Methods

### Plant growth conditions

Arabidopsis (*Arabidopsis thaliana*) *Ftsh11* knockout mutant *salk033047* and the corresponding Col-0 wild type (WT) used in this study for all Chl fluorescence measurements were obtained from the Arabidopsis Biological Resource Center (https://abrc.osu.edu). Arabidopsis seeds were suspended in 0.1% agar solution and cold-treated at 4 °C for 2 days. The vernalized seeds were sowed on well-moistened Sunshine 3 potting soil (Sun Gro Horticulture, Bellevue, WA) in Arabaskets placed in an Araflat (Lehle Seeds, Round Rock, TX). Each Araflat contained half Col-0 and half *salk033047* plants. The Araflats were covered with clear plastic covers for 5 day after planting to retain soil moisture and allow seed to germinate. Arabidopsis seedlings were thinned to 1 plant per Arabasket at 2 true-leaf stage and grown in a Reach-In Conviron growth chamber (E-15 model, Conviron, Winnipeg, Canada) for 3 weeks under optimal conditions (21 °C, 12 h/12 h d/n at 70 μmol quanta m^−2^ s^−1^). The plants were fertilized weekly with diluted Miracle-Gro nutrient (Scotts Miracle-Gro Products, Port Washington, NY). A total of six Araflats were planted for each experiments and the flats were rotated weekly.

### Treatments

For Chl fluorescence measurements, the Arabaskets with 21d–old, uniformly grown, WT and *salk033047* plants were selected and randomly rearranged into 4 treatment groups. 1) 21 °C control with normal light intensity (70 μmol quanta m^−2^ s^−1^), 2) 30 °C moderately elevated temperature with normal light intensity, 3) 35 °C elevated temperature with normal light intensity, and 4) 21 °C with high light (HL) intensity (400 μmol quanta m^−2^ s^−1^). The chambers used for the treatments were the same as the E15 Conviron model for growing the plants. Light intensity in each chamber was adjusted to the aforementioned levels using a light meter, and temperature settings in all chambers were checked with a thermocouple. The plants in groups 2 and 3 were placed into the heat treatment chambers with temperatures set at 30 °C or 35 °C, respectively, and light intensity was the same as that of optimal control conditions. The plants in HL treatment group 4 were placed to a 21 °C chambers with light intensity set at 400 μmol quanta m^−2^ s^−1^ whereas plants in group 1 remained in the original chamber. The photoperiod for all groups was the same 12 h/12 h light/dark cycle.

### Chlorophyll fluorescence measurements

The most recent fully-expanded intact rosette leaf from each Arabidopsis plant was used for Chl fluorescence measurements after 2 h dark-adaption. Dark treatment was applied by placing the enclosed leaf clips on to the selected rosette leaves. The fluorescence slow induction curve (IC) and the light response curve (LC) of the intact leaves were measured [45] at the onset of the treatment (0 day) and then daily for 5 days (1, 2, 3, 4, and 5 days) using a pulse amplitude modulation fluorometer equipped with a dual wavelength emitter detector unit (DualPAM 100, Walz, Effeltrich, FRG). At the set treatment times, the 2 h dark-adapted leaf together with the enclosed leaf clip on was carefully removed from base of the petiole and then immediately mounted on the measuring head of DualPam100 system under dim light**.** At the beginning of each measurement, actinic light (20 Hz) was provided to measure the minimal (F_o_) and maximal (F_m_) Chl fluorescence of the dark-adapted leaf tissues and to determine the maximum quantum efficiency [F_v_/F_m_ = (F_m_-F_o_)/F_m_] of PSII photochemistry. The *F*_v_/*F*_*m*_ was automatically calculated where *F*_v_ represents the maximum variable fluorescence (F_v_ = F_m_-F_o_). Forty seconds after the onset of actinic light, a saturating pulse (SP) was given in 20 s intervals over a 5-min period for IC measurements. The SP had an intensity of 6000μE m^−2^.s^−1^ at an emission peak of 660 nm and pulsed for 1 s. For the LC measurement, the red actinic light was automatically applied in a stepwise increasing in photosynthetically active radiation (PAR) from 0 to 8, 15, 24, 55, 97, 128, 218, 341, 533 and 827 in 30 s intervals over a 5-min period using a DualPam100 system. Fluorescence parameter changes (response curves) of the leaf tissues in response to the SP (IC) and light intensity increases (LC) were recorded over each measurement [46]. The changes of the P700 redox state of PSI were recorded during the IC at an absorbance wavelength of 830 nm. For each treatment, leaves from 6 to 8 plants were used for daily Chl fluorescence measurements. The experiment was repeated 4 times. Information about the Chl fluorescence parameters reported here and equations for their calculation are provided as the supplementary material (Additional file 1: Table S1).

## Results

### The maximum quantum efficiency of PSII (F_v_/F_m_) decreased significantly in *FtsH11* under elevated temperatures

**Fig. 1:**
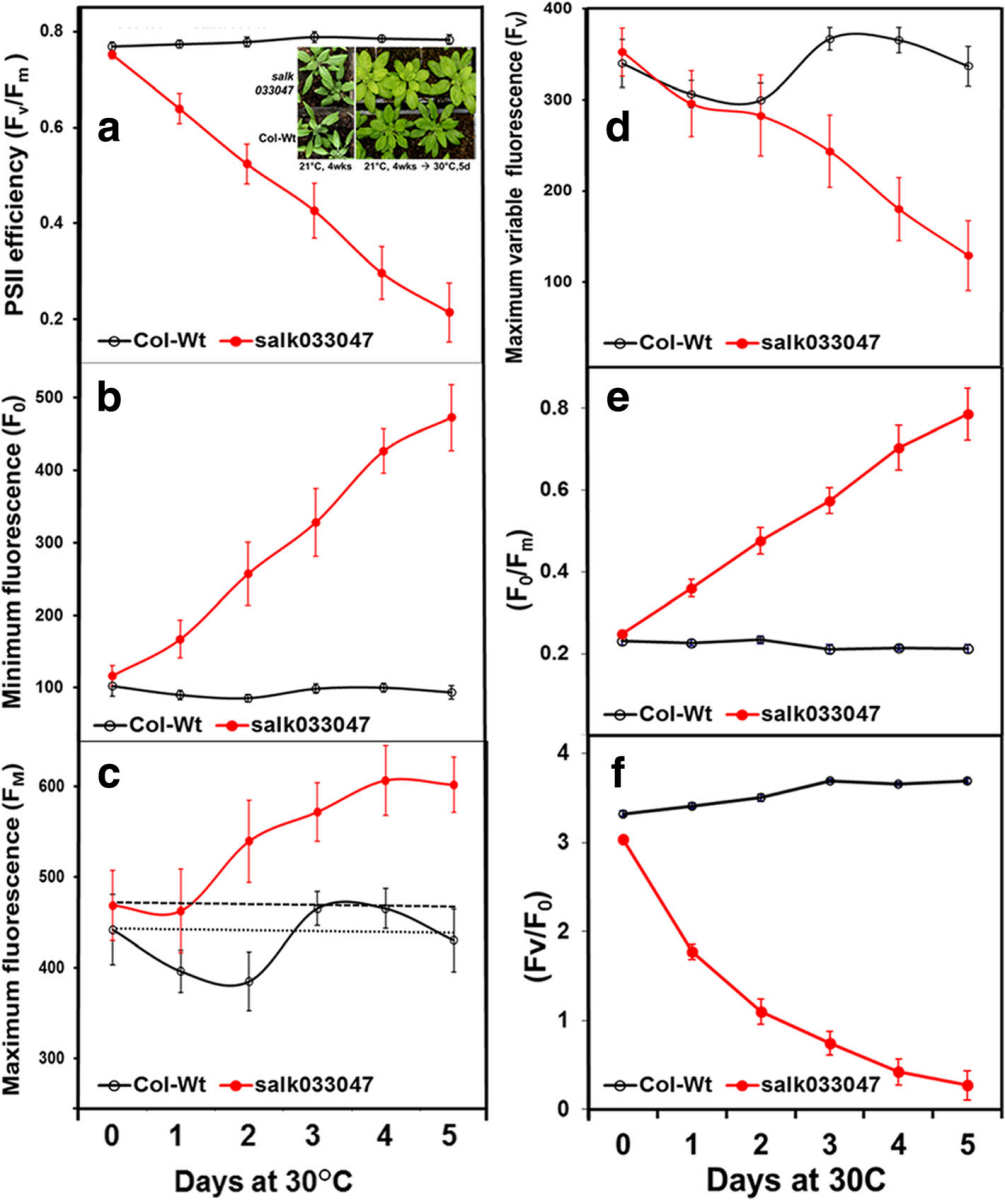
Changes in chlorophyll fluorescence in dark-adapted leaves under moderately elevated temperature at 30 °C. **a** the maximal PSII efficiency (*F*_v_/*F*_*m*_), (**b**) maximal Chl fluorescence (F_m_), (**c**) minimal Chl fluorescence (F_o_), (**d**) maximal variable Chl fluorescence (F_v_), (**e**) the correlation of F_m_ and F_v_ with F_o_ and (**f**) the daily *F*_*v*_*/F*_*o*_ ratio changes in Col WT and (*salk033047*) *FtsH11* mutant over a 5 day 30 °C-treatment. The first data point in (**a**) and the data point at the 0d represent the data collected from 21 °C-grown plants prior to the onset of heat treatment. Three-week old Col WT and *FtsH11* mutant (*salk033047*) plants grown at 21 °C 70 μmol with 12 h/12 h d/n cycle were transferred to a 30 °C growth chamber. Changes in dark-adapted chlorophyll fluorescence were determined daily for five days. For each sampling point, the most recently matured leaves from 6 to 8 plants were used. The experiments were repeated 4 times. Each data point represents mean ± SD

**Fig. 2:**
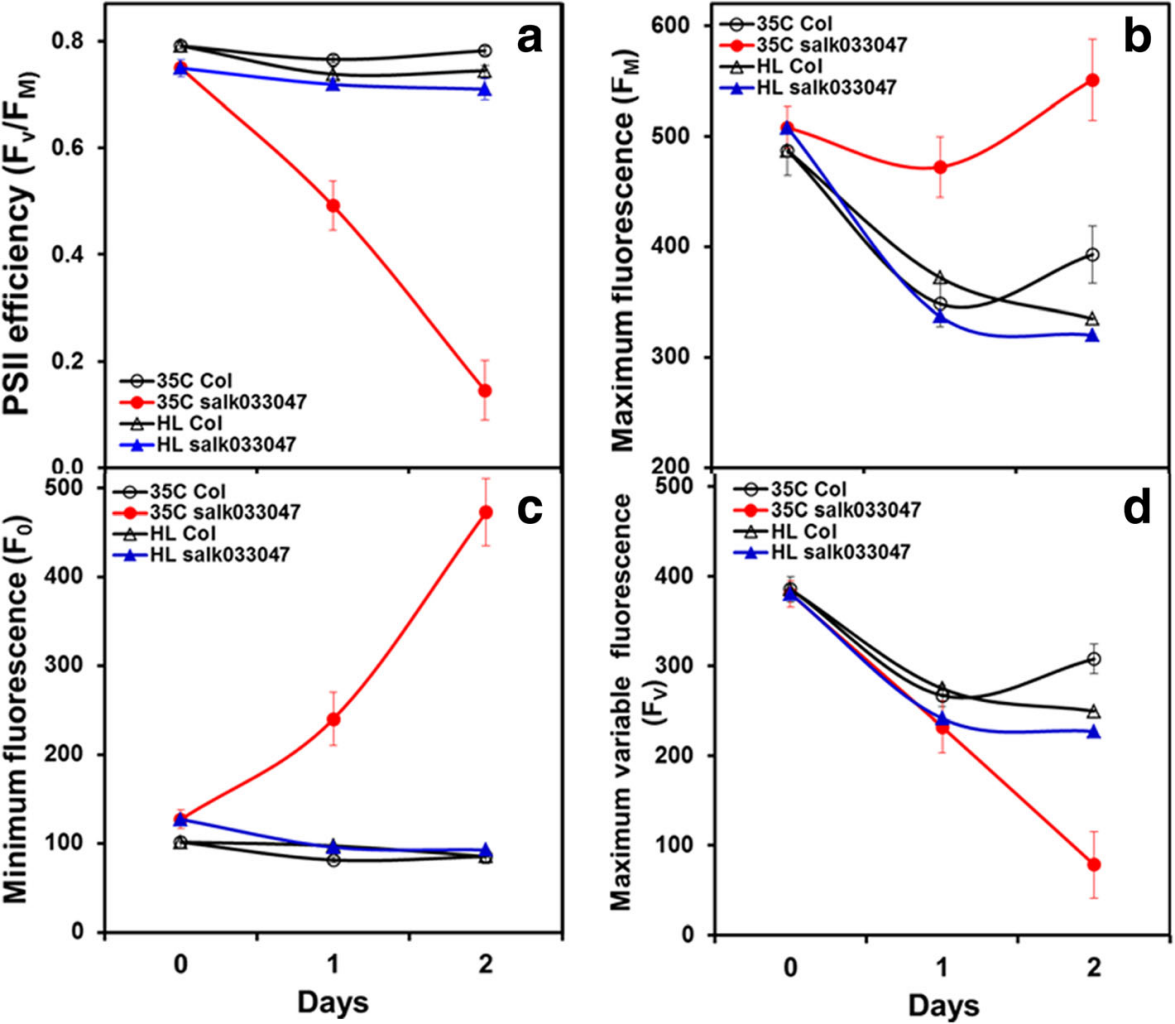
Changes in chlorophyll fluorescence of dark adapted leaves of Col WT and *FtsH11* mutant (*salk033047*) under moderately high temperature at 35 °C or a 2d high light treatment (HL, 400 μmol quanta m^−2^ s^−1^) at normal temperature (21 °C). **a** the *F*_v_/*F*_*m*_, (**b**) F_m_, (**c**) F_o_, and (**d**) F_v_ values in rosette leaves were measured daily after 2 h of dark adaption

Under normal 21 °C growing temperature, no significant difference was detected in the measured Chl fluorescence parameters between the dark-adapted *Ftsh11* mutant *salk033047* plants and WT Col-0 plants (Figs. 1 and 2, 0d). At 21 °C, *F*_v_/*F*_*m*_ of *salk033047* and Col-0 plants were 0.769 and 0.752, respectively (Fig. 1a, 0 d). Highlight (400 μmol quanta m^−2^ s^−1^) at 21 °C had no significant effect on *F*_v_/*F*_*m*_ in both *Ftsh11* mutant and WT plants (Fig. 2a). However, after subjecting to elevated temperatures of 30 °C and 35 °C, the *F*_v_/*F*_*m*_ of mutant plants decreased rapidly over the course of high temperature treatments (Figs. 1 and 2a). At 30 °C, the *F*_v_/*F*_*m*_ of *salk033047* decreased from 0.752 at 0d (21 °C) to 0.639, 0.524, 0.426, 0.297 and 0.214 after 1d, 2d, 3d, 4d, and 5d of heat exposure, respectively (Fig. 1a). The average reduction of *F*_v_/*F*_*m*_ was −0.109 per day with a R^2^ of 0.998. At 35 °C, *F*_v_/*F*_*m*_ of the mutant plants decreased from 0.749 (0d) to 0.492 after only 1d of heat exposure and it plummeted to 0.145 at the end of 2d treatment (Fig. 2a). Therefore, the 35 °C treatment was terminated after 2 days. The average reduction of *F*_v_/*F*_*m*_ was −0.302 per day in 35 °C-treated *salk033047* plants with a R^2^ of 0.993, much faster than that occurred at 30 °C.

### Changes in individual Chl fluorescence parameters in *FtsH11* under elevated temperature conditions

As indicated by the way the *F*_v_/F_m_ was calculated [(F_m_ – F_o_)/*F*_*m*_], the decreases in *F*_v_/F_m_ in heat-treated *FtsH11* mutant could be a result from either an increase in F_o_ or a decrease in F_m_ or a combination of both (decrease in maximal variable fluorescence F_v_). To determine the cause for *F*_v_/*F*_*m*_ decrease in *salk033047* and its association with *Ftsh11* mutation, we examined the kinetic responses of each individual Chl fluorescence parameter to the applied heat treatments in mutant plants and compared with those in WT under the same conditions.

Upon exposure to moderately heat treatments, F_o_ of *salk033047* plants displayed a dramatic increase while it showed no significant change in Col-0 plants under both elevated temperatures. After only 1 day of exposure to the 30 °C treatment, the F_o_ of *salk033047* plants increased substantially (Fig. 1b). The F_o_ of mutant plants increased continually with heat exposure time, whereas F_o_ of WT plants showed little change under the same treatment (Fig. 1b). The rate of F_o_ increase in 30 °C-treated mutant plants averaged 75.2 per day with a R^2^ of 0.992. The average F_o_ in 5d, 30 °C-treated mutant plants increased more than 4-fold from 116 to 472 (Fig. 1b). The increase in F_o_ accelerated further after exposing mutant plants to a 35 °C heat treatment, at a rate (172.8/day) that was twice of 30 °C-treated mutant plants (Fig. 2b). The F_o_ of 2d, 35 °C-treated *salk033047* plants exceeded more than 4-folds of 21 °C-controls (0d). The differences of F_o_ levels were highly significant not only between mutant and WT plants that underwent the same treatment and between control and heat-treated mutant plants, but also between the 30 °C and 35 °C treated mutant plants (Fig. 1 and 2b).

Adjustments for F_m_ were observed in both WT and mutant plants within the first day exposure to either moderately elevated temperatures or HL treatment (Figs. 1 and 2c). F_m_ displayed an initial decrease followed by an increase during the course of heat treatments. Although the kinetic change of F_m_ in mutant plants resembled those exhibited by WT in response to the heat treatments, the degrees of initial decrease in heat-treated *salk033047* was considerable less than those seen in WT plants (Fig. 1 and 2c). The F_m_ levels of heat-treated mutant plants were also much higher than those of WT plants. The F_m_ of 2d and 4d 30 °C-treated mutant plants were 539 and 606 respectively comparing to 385 and 465 in heat-treated WT (Fig. 1b). The differences in F_m_ between mutant and WT leaves became significant after 2 days of 30 °C treatment (Fig. 1c). The difference in F_m_ was widened further between mutant and WT plants under 35 °C condition (Fig. 2c).

F_v_ is the difference between F_m_ and F_o_ and represent the maximum variable fluorescence for photochemical quenching of PSII. After the onset of the heat treatments, the F_v_ of *salk033047* plants showed a steady decrease (Figs. 1 and 2d), despite of the detected F_m_ increases (Fig. 1 and 2c). The F_v_ of 30 °C-treated *salk033047* plants showed a clear negative correlation with F_o_ (Fig. 1e). The similar correlation relationship was also observed in 35 °C-treated mutant plants (data not shown). The moderate increases in F_m_detected in heat-treated mutant plants (Fig. 1b) was simply unable to compensate for the effect of heat induced F_o_ increase for F_v_ (Fig. 1e). Therefore, the decreases in F_v_ of *salk033047* plants resulted solely from the drastic increases in F_o_ occurred under heat treatment conditions (Fig. 1f). Consequently, the heat induced increases in F_o_ primarily resulted in the significant decrease in *F*_v_/F_m_ in heat-treated *FtsH11* mutant plants (Figs. 1 and 2).

### The *FtsH11* mutant displayed similar Chl fluorescence as the WT in response to high light stress at 21 °C

To determine if the *Ftsh11* mutation also affects the response of Arabidopsis to HL stress, we measured the Chl fluorescence yields of *salk033047* and Col-0 under a light intensity about five folds higher than optimal level at normal 21 °C. We found that, in response to a light intensity increase from 70 μmol quanta m^−2^ s^−1^ to 400 μmol quanta m^−2^ s^−1^, the F_m_ of *salk033047* and Col-0 mutant plants decreased within the first day of HL exposure but exhibited no changes in F_o_ (Fig. 2). The F_v_ changes in HL-stressed mutant and WT basically followed the same pattern of F_m_ changes (Fig. 2c and d). As a result, no HL induced net decreases in *F*_v_/*F*_*m*_ were detected in either plant line. Overall, the HL-stressed *salk033047* and Col-0 plants showed no significant differences in F_o_ F_m_ F_m_, and *F*_v_/*F*_*m*_ over the period of 2d treatment (Fig. 2), indicating that mutation of *Ftsh11* did not affect the adaption processes of *salk033047* leaves to high light stress in Arabidopsis under normal temperature.

### The electron transfer rates (ETR) of PSII and PSI decreased significantly in *Ftsh11* mutant at 30 °C

**Fig. 3:**
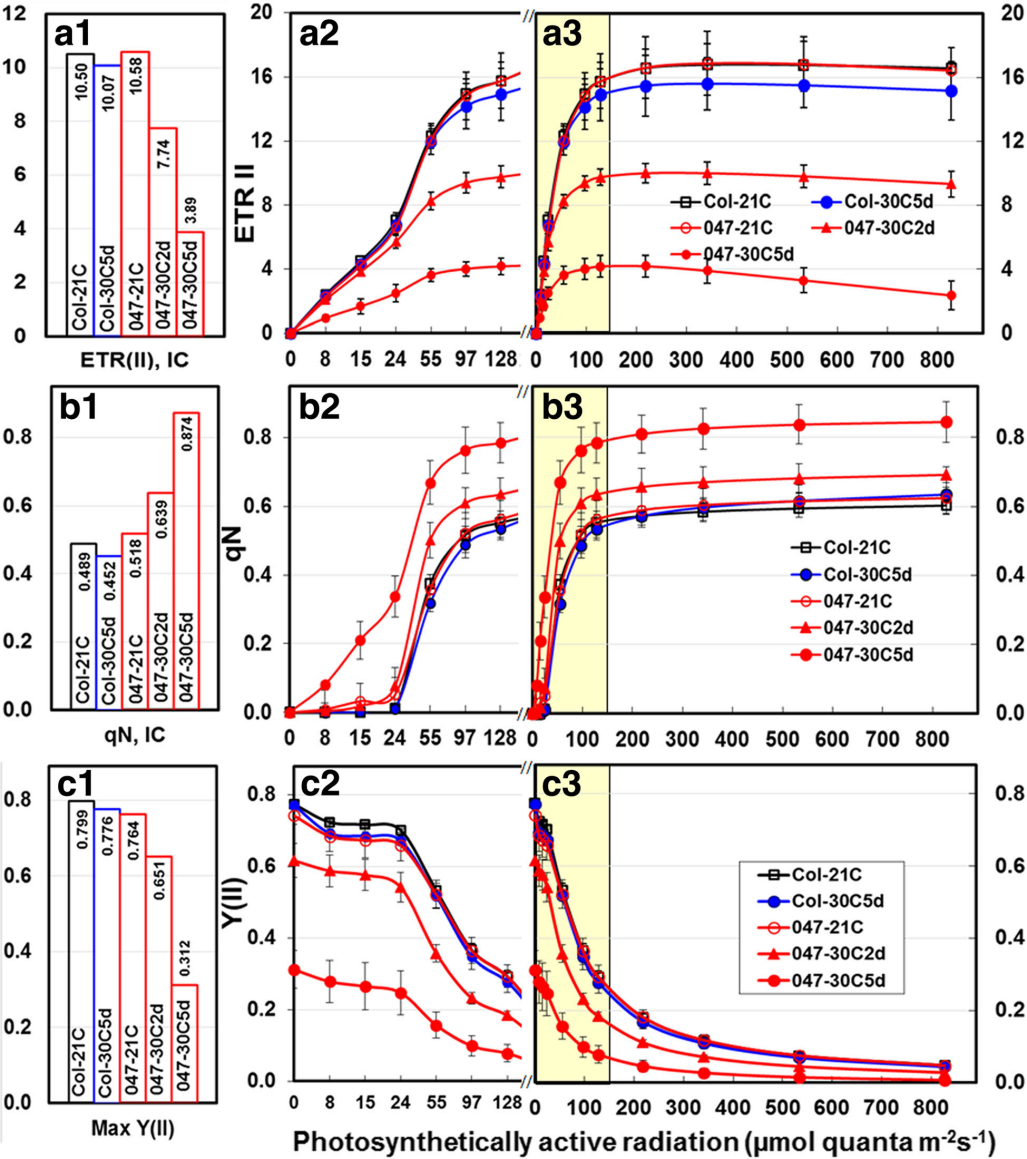
Light response curve measured in light adapted leaves in 21 °C, and 30 °C5d Col WT and in 21 °C, 30 °C2d and 30 °C5d treated *FtsH11* mutant (047) plants. **a** Electron transfer rate of PSII, ETR(II); (**b**) Coefficient of nonphotochemical quenching of PSII, qN; (**c**) Effective quantum yield of PSII, Y(II). The maximal levels of the corresponding parameters (number inside the graph) obtained in IC measurement are presented here as fig. *a1*, *b1* and *c1*. The X axis for fig. *a2*, *b2* and *c2* was plotted with even interval for the first six low light intensities measurement to provide readers a better view for the data points at lower light intensity range. The corresponding data points were shaded in fig. *a3*, *b3*, and *c3*. For each sampling point, 6–8 plants were used. The experiments were repeated 4 times. Each data point represents mean ± SD. Here, the mutant *salk033047* was written as 047 due to space restraint

**Fig. 4:**
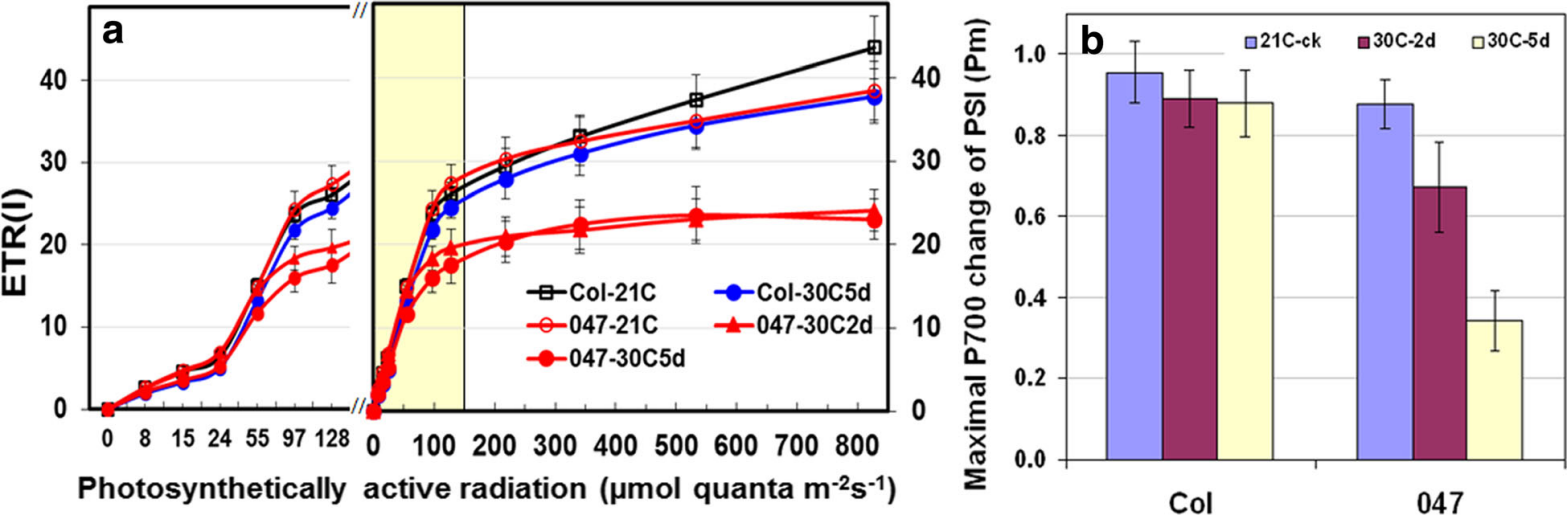
Light response curve of electron transfer rate of PSI, ETR(I) (**a**) and maximal P700 of PSI, Pm (**b**) measured in light adapted Col WT and *FtsH11* mutant (047) plants at normal 21 °C, 2d and 5d of 30 °C treatment

The IC and LC measurements were used to further dissect the effects of *FtsH11* mutations on photosynthetic systems under moderate heat stress. Significant differences in a range of measured parameters were detected between the heat-treated *salk033047* and Col-0 plants and between heat-treated and 21 °C-control *salk033047* plants. No differences in the measured parameters were detected between control *salk033047* and Col-0 and between heat-treated and control WT plants. The LC of ERT(II), qN and Y(II) and the F_m_, maximal levels of ERT(II) and qN of PSII obtained in the IC in control (0d) and 2d, and 5d 30 °C-treated *salk033047* and control and 5d 30 °C Col-0 plants are presented in Fig. 3. The ETR(I) and maximal P700 of PSI **(**Pm) are presented in Fig. 4. The kinetic responses of major Chl fluorescence parameters for PSII and PSI in dark-adapted plants obtained from IC are presented in Additional file 2: Figure S1. A table summarizing the responses of all parameters of IC and LC is provided as supplemental materials (Additional file 1: Table S1).

In 30 °C-treated *salk033047*, significant decreases in ETR (II) were detected at all light intensity applied in 5d–treated plants and at 55 PAR and above light intensity in 2d–treated plants (Fig. 3a). At the onset of light induction 8 PAR, the ETR(II)s were 2.08 and 0.94 in 2d and 5d–treated mutant comparing to the 2.24 and 2.41 measured in 21 °C-contrl mutant and 5d–treated WT. Such ETR(II) difference expanded as the applied light intensity increased over the course of LC measurement. The levels of heat induced ETR(II) decreases in *salk033047* plants also showed an apparent correlation with the heat exposure times. The ETR(II)s in 5d–treated *salk033047* were not only significantly lower than those of heat-treated Col-0 plants, but also were significantly lower than those of 2d–treated mutant plants (Fig. 3a). At 97 PAR, the average ETR(II)s in 2d and 5d treated *salk033047* plants were 9.38, 4.01 respectively, comparing the 14.17 in 5d–treated Col-0. At 827 PAR, ETR(II)s changed to 9.33, 2.34 and 15.17 in these corresponding plants. The IC measurements detected similar ETR(II) changes in heat-stressed *FtsH11* mutant (Additional file 2: Figure S1). At the end of IC illuminations, ETR(II)s of 2d and 5d treated *salk033047* were 7.74, 3.89, respectively, comparing 10.58 in control mutant and 10.07 in 5d treated WT (Fig. 3a).

The response of ETR(I) of PSI in *FtsH11* mutant to the 30 °C heat treatment were similar to those of ETR(II), except that the exposure to 30 °C beyond the 2nd day did not induce further decrease in ETR(I) of the mutant plants (Fig. 4a). The results indicate that mutations of FtsH11 in Arabidopsis reduce the electron transfer efficiency at elevated temperatures.

### Nonphotochemical quenching (NPQ) increased significantly in *Ftsh11* mutant at 30 °C

The coefficient of NPQ, qN, was used to evaluate the effects of *FtsH11* mutation on the efficiency of PSII in the energy dissipation in Arabidopsis chloroplast. The higher the qN, the higher proportion of the absorbed light energy being dissipated (quenched) into heat, indicates the less efficiency of PSII in the use of excitation energy for photochemical reaction. In both IC and LC measurements, we detected increases in qNs in the *FtsH11* mutant after subjecting the plants to the 30 °C treatment (Fig. 3b, Additional file 2: Figure S1). Opposite to what occurred in the 5d–treated WT (a slight delayed response) the qNs in 5d–treated mutant plants raised much fast after the onset of induction and were substantially higher than those of WT plant as well as those of 21 °C-grown mutant plants (Fig. 3b). The qNs of 5d–treated mutant plants were also significantly higher than those of 2d, treated mutant plants, indicating a positive correlation between the qN increases of mutant plants and the heat treatment times. At 827 PAR, the measured qNs in 2d and 5d treated *salk033047* plants were 0.692 and 0.845, respectively comparing 0.625 in control mutant and 0.635 of 5d–treated Col-0 plants (Fig. 3b). The highest qN obtained in IC were from either 5th or 6th SP, with relative values of 0.639, 0.874, 0.518 and 0.452 for the corresponding plants (Fig. 3b1).

### The effective quantum yield of PSII (YII) and maximal P700 of PSI (Pm) decreased significantly in *Ftsh11* mutant under moderately elevated temperatures

Both IC and LC measurements detected reductions of YII in 30 °C-treated *FtsH11* mutant. Consistent with the *F*_v_/F_m_ change presented above, the YIIs of *salk033047* were significantly lower than those of heat treated Col-0 WT and 21 °C-control plants (Fig. 3c). Again, here we observed a positive correlation between the heat exposure times with the degree of the YII reduction in *salk033047* plants (Fig. 3c). We also observed high light induced decreases in YII in WT and mutant plants in LC measurements (Fig. 3c), a phenomenon called photoinhibition. However, the responses of *salk033047* plants to the photoinhibition were remarkably similar to those of WT plants and showed no association with the heat treatment (Fig. 3).

The IC and LC measurements also revealed significant decreases in the maximal P700 of PSI (Pm) in 30 °C-treated *salk033047* plants (Fig. 4b). The decreases in Pm of mutant plants also showed an apparent correlation with the duration of the heat exposure (Fig. 4b). The longer the heat treatment, the larger decreases in Pm of mutant plants. Furthermore, moving the 21 °C-grown plants to 25 °C also induced a moderate reduction in Pm of mutant plants even though no changes in PSII efficiency of mutant plant were detected at this temperature (Additional file 3: Figure S2). Also, the Pm of control *salk033047* plants (0.867) was slightly lower than Pm of control Col-0 (0.949), much similar to that (0.889) of 5d–treated WT (Fig. 4b).

## Discussion

### FtsH11 protease play an essential role in maintaining photosynthetic efficiency of photosystems at elevated temperatures in *Arabidopsis*

The FtsH11 is a chloroplast targeted protease [25] located exclusively in the chloroplast envelope [47]. It is the first plant FtsH that has been shown to play an essential role in thermotolerance in Arabidopsis [16], while most other well-studied chloroplast FtsH proteases functions in protecting the photosystems from high light stress [14, 15, 48]. Mutations in FtsH11 cause significant decreases in photosynthetic capability and a bleaching chlorotic leaf phenotype [16]. Similar chlorotic leaf phenotype has been observed in *ftsh11* when grown under natural sunlight in the field in northern Sweden [28] and under continuous light at relatively high light intensities [49], conditions that are known to induce photoinhibition and cause damages to PSII [50–52]. Hence, a role of FtsH11 in protecting PSII from light stress has also been proposed [49]. Because high temperatures, even moderately high, can induce biochemical and structural changes and cause damages to PSII very much like those of high light stress [4, 53, 54], we used experimental conditions that can distinguish the changes in PS performance induced by heat from those induced by HL in *FtsH11* mutant in this study. The light condition used for growing the plants and for the heat treatments was at the low end of optimal light condition (70 to 100 μmol quanta m^−2^ s^−1^) for Arabidopsis plant (12 h/12 h d/n) and generally do not impose light stress to photosystems of normal Arabidopsis plant. Likewise, the HL treatment performed at about 6 folds higher light intensity than of control but normal 21 °C temperature eliminated the likelihood of heat stress imposing on photosystems in Arabidopsis plant. Therefore, the differences detected between heat-stressed (30 °C and 35 °C) and control (21 °C) plants under normal light are likely to be caused by the moderately elevated temperatures while those detected between HL-stressed and normal light grown plants at 21 °C are likely to be induced by light stress. Under all the treatment conditions, we detected significant decreases in both *F*_v_/*F*_*m*_ of PSII and Pm of PSI only in moderate heat-stressed *ftsh11* plants while no differences were shown under either the control or HL-stress condition between the WT and *FtsH11* mutant (Figs. 1, 2, 3 and 4). In plants, *F*_v_/*F*_*m*_ and Pm are photosynthetic parameters indicating an overall photochemical efficiency of PSII and PSI, respectively. Decreases in *F*_v_/*F*_*m*_ and Pm under stress conditions are indicators of stress induced damages to PS [55], mostly occurred at the reaction centers (RC) and the light harvesting complexes (LHC) [5, 40]. The specific association of *F*_v_/*F*_*m*_ and Pm decreases with FtsH11 mutation under moderate temperatures suggests a primary function of this protease in maintaining photosynthetic activities of both PSII and PSI as the environmental temperature elevated above optimal. The correlation relationship between the degrees of *F*_v_/*F*_*m*_ and Pm decreases in *FtsH11* mutant and the durations and levels of the heat stress applied revealed in this study (Figs. 1a, 2a and 3c) further imply the active involvement of the *FtsH11* in the adaptation and/or protection of the photosynthetic systems to/from heat stress in Arabidopsis. Furthermore, the study also revealed a negative correlation between the increases in F_o_ and the decreases in F_v_ during the course of heat treatment in mutant plants (Fig. 1e), therefore, uncovering the main contributing factor (F_o_) to the detected decreases in *F*_v_/*F*_*m*_ in *ftsh11* (Figs. 1 and 2).

### The role of *FtsH11* in regulating the adaptation of photosystems to temperature increases in *Arabidopsis*

The present study has shown that the mutation of *FtsH11* caused alterations in the array of Chl fluorescence parameters under moderately elevated temperatures of 30 °C and 35 °C (Figs. 1, 2, 3 and 4). The results reveal several lines of evidence for a critical role of FtsH11 in maintaining the overall structural and/or biochemical stability of photosynthetic systems at moderately elevated temperatures. First, the gradual but significant increase in F_o_ occurred only in heat stressed *FtsH11* mutants, indicating damages to PSII RCs in *FtsH11* plants under moderately high temperatures (Figs. 1 and 2c). The F_o_ in the dark adapted plant represents the dark fluorescence yield where all RCs of PSII are open. It has been shown that structural and biochemical alternations to the PSII RCs, such as dissociation of LHC from RC and monomerization of LHC can increase F_o_ [56, 57]. The increases in F_o_ have also been associated with PSII damages caused by both high temperature and high light stresses in plants [58]. The level of F_o_ is known to be closely associated with the thermostability of PSII and has been used to examine the damages to PSII caused by high temperature [42, 43]. High temperature induced severe structural and function damages to PSII is indicated by the significant increase F_o_ in Chl fluorescence measurements [42, 43]. Normally, the rapid increase of F_o_ occurs at temperatures about 40 °C or above, ranging from 40 to 50 °C depending on plant species, indicating severe damage to PSII [41, 42, 45]. Here we detected significant increases in F_o_ in *FtsH11* mutant at 30 °C, a temperature that does not usually cause noticeable changes in PSII photochemistry, even for a cool season plants of Arabidopsis (Figs. 1, 2, 3 and 4). The results indicate severe damages to PSII LHC and RCs caused by either structural or biochemical alternations or a combination of both in PSII of *FtsH11* plants under moderately elevated temperatures of 30 °C. It is known that chloroplasts respond to temperature increases by adjusting membrane properties [59, 60], modification of the configuration and/or spatial organization of protein complexes on thylakoids, regulation of the turnover of specific proteins such as D1 protein of PSII [34], and repairing damaged proteins to maintain structural intactness and function normality [5, 54, 57]. The proteolytic activities mediated by various enzymes, including FtsH proteases, are essential for efficient photosynthesis and critical for maintaining stability of various complexes in chloroplasts in response to daily temperature and light fluctuation and under high temperature and radiation stress [26, 31, 32, 51, 61]. The significant F_o_ increases in *FtsH11* under moderately elevated temperatures indicates that FtsH11 protease plays essential roles in maintaining thermostability of photosystems in response to temperature increases. Moreover, no significant changes in F_o_ at HL condition and no significant differences in other Chl fluorescence parameters between WT and *FtsH11*mutant plants under control and HL conditions suggest that *FtsH11* function specifically in maintaining thermostability of PSII under high temperature conditions. Therefore, maintaining a normal function of *FtsH11* in chloroplast could be vital for plant’s adaptability to the daily and seasonal temperature fluctuation.

Second, the significant increases in qN of heat-treated *FtsH11* plants (Fig. 3b) implied that the mutation in *FtsH11* had a negative effect on the PSII photochemical efficiency in using the absorbed energy when growth temperature increased above optimal. The qN is a Chl fluorescence parameter commonly used to describe the efficiency of PSII in energy dissipation. In plants, the increase in NPQ often occurs when the light intensity of the growth environments exceeds the capacity for photosynthesis [5, 62]. It is a protective mechanism of plants to dissipate excess absorbed light energy as heat in order to prevent the generation of reactive oxygen species (ROS) and photodamages to photosystems (especially PSII) under high light condition [5, 62]. NPQ is a sensitive parameter for monitoring damage to photosystems caused by various environmental stresses [37, 40, 55]. Conformational changes of the thylakoid membrane, unstacking of grana and conformation change, monomerization of LHCII, and damages to PSII-LHCII induced by environmental factors such as high temperature and/or high light, are thought to be major causes for NPQ increases [57, 61, 63–65]. Although photosystems of *FtsH11* plants appeared to function similarly as those of WT plants under normal temperature, the significant qN increase in *FtsH11* after exposing to 30 °C (Fig. 3 and Additional file 2: Figure S1) indicates that the impairment of photosystems in utilizing the absorbed light energy for photochemistry in heat stressed mutant plants. The correlation of qN increases with the heat exposure times further point to the heat induced damages to photosystems in mutant plants. The results revealed an important role of *FtsH11* protease in regulating functional stability of photosystems in response to environmental temperature increases in Arabidopsis.

Third, the detected decreases in ETR provide direct evidence on the effect of *FtsH11* mutation on electron transport in both PSII and PSI (Fig. 3). High temperature stress induced changes in ETR have been reported in sweet sorghum and other species [5, 39, 44, 53]. It is known that increases in temperatures not only increase thylakoid membrane fluidity but also cause rearrangement and even disassociation of protein complexes and photosystems [4, 5]. Extreme heat induced structural changes in protein complexes, damage to photosystems, and the loss of oxygen evolving activity all reduce the ability of photosystems in electron transport [4, 53]. Here we showed significant ETR(II) and ETR(I) decreases in *FtsH11* under a moderately elevated temperature which caused no ETR change in WT plants (Figs. 3 and 4). The results demonstrate the involvement of *FtsH11* protease in maintaining effective electron transport of photosystems as environmental temperature increases in Arabidopsis.

In most studies, heat stress induced drastic functional and structural changes of photosystems often occur at extreme temperatures [39, 42, 44]. For most plants, it happens at mid to upper 40s°C [36, 43, 44, 66]. For cool-season species, such as Arabidopsis, wheat and barley, it occurs at temperature of about 40 °C to lower 40s [41, 42, 45]. The results of WT plants presented in this study show normal photosynthetic activities of Arabidopsis plants at treatment temperatures of 30 °C and 35 °C (Figs. 1, 2, 3 and 4, Additional file 2: Figure S1). The results of this study (Figs. 1, 2, 3 and 4, Additional file 2: Figure S1), however, reveal drastic changes for a range of Chl fluorescence parameters in *FtsH11* mutant at 30 °C, a temperature much lower than typically reported temperature that alter the efficiency of photosynthetic systems. The findings all point to severe damages to photosynthetic systems in *FtsH11* plants under this moderately elevated temperature at which WT plants functioned normally. The fact that photosynthetic systems of *FtsH11* mutant functioned similarly as those of WT plants at optimal temperature under either normal light or high light conditions (Figs. 1, 2, 3 and 4) suggests specific role of FtsH11 in the maintenance of functional and structural stability of photosynthetic systems as the growth temperature elevated above optimal. The F_o_ increases in *FtsH11* at 30 °C and 35 °C are likely the result of heat-induced dissociation of light harvesting complex (LHC) from the reaction center complexes and/or monomerization of LHC, reported phenomenon observed under extreme high temperature conditions [64]. The significant increases in qN and decreases in *ETR(II)* and YII of PSII and *ETR(I)* of PSI in 30 °C-treated *FtsH11* plants are likely to be the results of heat induced damages to photosystems and possibly heat-induced configuration changes of protein complexes on thylakoid membranes.

### The FtsH11 mediated thermotolerance of photosynthetic systems appears to be independent from oxidative stress

It is well known that heat stress induces the production of ROS such as hydrogen peroxide and superoxide [5]. Bursts of ROS (oxidative stress) can inhibit the PSII repair processes and the turnover of damaged protein subunits of PSII [52]. Using spinach thylakoids, Yamashita and colleagues have showed that the heat inactivation of PSII is caused by ROS produced during moderate heat stress processes [67]. Some of the Chl fluorescence parameter changes observed in the 30 °C-treated *FtsH11* plants resemble those reported in oxidative stressed plants (Figs 1 and 2). Therefore, the possible association of the detected changes in Chl fluorescence in 30 °C-treated *FtsH11* with overproduction of ROS was examined. The accumulations of hydrogen peroxide and superoxide in the most recent expanded rosette leaves of mutant and WT plants were compared by nitroblue tetrazolium and 3,3′-diaminobenzidine staining, respectively. Surprisingly, we found no noticeable difference in the level of these ROS accumulation (Additional file 4: Figure S3). The result also showed very minimal ROS induction under the moderate heat condition used in this study, thus rule out the possibility of ROS accumulation as a cause for the phenotypic and Chl fluorescence changes observed in heat-treated *FtsH11* plants. Therefore, the thermotolerance mechanism of photosynthetic systems mediated by *FtsH11* protease in chloroplasts is likely to be independent of oxidative stress.

### The increase in F_o_ in *FtsH11* mutants was not affected by HCF genes

The high-chlorophyll-fluorescence mutants (*hcf*) have been known to have high F_o_ value [68, 69], a phenotype similar to that observed in *ftsh11* plants under moderately elevated temperatures (Figs. 1 and 2c). To determine the possible association of *FtsH11* with any of the *HCF* genes as well as their association with moderate heat stress, we measured the F_o_ and *F*_v_/F_m_ levels in all Arabidopsis *HCF* mutants available in ABRC (http://www.arabidopsis.org/) over a 5 d, 30 °C treatment. Although the F_o_ levels of all *HCF* mutants were higher than those of Col-0 WT and *ftsh11* plants under normal 21 °C temperature, no further increase in F_o_ was observed in any of *HCF* mutants under the 30 °C treatment (Additional file 5: Table S2). Also, unlike *FtsH11* mutants, we detected no significant decreases in *F*_v_/F_m_ in 30 °C-treated *HCF* mutants. All leaves of *HCF* mutants stayed green in color and chlorophyll content remained at the same level throughout the course of 30 °C treatment. These results suggest that FtsH11 protease mediated mechanisms in maintaining normal photosynthetic activities at moderately elevated temperatures are likely to be independent from the functions of those *HCF* genes examined in this study.

## Conclusions

This study shows that mutations in the *FtsH11* protease gene altered an array of Chl fluorescence parameters, resulting in significant reduction in photosynthetic efficiency of PSII and PSI within a few days of a 30 °C challenge. It also reveals that the drastic F_o_ increases contributed the most to the detected decreases in maximum quantum efficiency of PSII photochemistry of *FtsH11* mutant plants at elevated temperatures. The findings of this study show that the *FtsH11* protease mediated network is essential in maintaining normal photosynthetic activities of photosystems possibly via its regulatory functions in the thermostability of photosynthetic machineries as environmental temperature rises above optimal. Such roles of *FtsH11* protease appear to be specific to the adaptive mechanism of photosystems to temperature increases in chloroplasts. Disruption of the *FtsH11* renders *FtsH11* plants unable to perform these required regulatory functions in chloroplast. To understand the network regulated by *FtsH11*, it is necessary to identify the substrate of *FtsH11* and the downstream components using genetic, biochemical and molecular approaches. To translate the discovery from Arabidopsis, the *FtsH11* homologs should be evaluated in crop species for their functions in alleviating negative impacts of heat stress to photosystems. These experiments will offer new insights into the proteolytic processes of *FtsH11* in regards to maintaining efficient photosynthesis under moderate heat stress and a foundation for improving photosynthetic efficiency of crop plants, hence sustain agricultural productivity under unfavorable environments.

## Additional files

Additional file 1: Table S1. Summary of the IC and LC chlorophyll fluorescence parameters in Col-0 WT and *FtsH11* mutant *salk033047* plants in response to the 30 °C-treatment. Definition for the Chl fluorescence parameters and their calculation equations are provided in the second sheet. (XLSX 19 kb)

Additional file 2: Figure S1. Induction response curves of chlorophyll fluorescence parameters of PSII and PSI in dark adapted Col-0 WT and *salk033047* mutant (047) plants (plots with the “Time” as X-axis units). The light response curves of the Y(II), Y(NPQ), and Y(NO) of light adapted plants was also included here (plots with the light intensity “PAR” as the X-axis unit). (TIFF 9702 kb)

Additional file 3: Figure S2. Measured P700 of PSI (Pm) and photosynthetic efficiency of PSII (YII) changes in 2 and 5 day, 25 °C treated Col WT and *FtsH11* mutant *salk033047* (047) leaves. The 25 °C-treatet mutant plants showed marginal decreases only for Pm in dark adapted plants and PSI and PSII activities were determined immediately on DualPAM100. (TIFF 2259 kb)

Additional file 4: Figure S3. Accumulation of ROS in rosette leaves of 21 °C-control and 30 °C-treated Col-0 WT and *FtsH11* mutant *salk033047* plants. The leave tissues were harvested and stained in nitroblue tetrazolium (NB) and 3,3′-diaminobenzidine (DAB) solution to examine the production of hydrogen peroxide and superoxide respectively. (TIFF 1831 kb)

Additional file 5: Table S2. **A**) Comparisons of minimum chlorophyll fluorescence yield (F_o_) of high chlorophyll fluorescence (*hcf*) mutant with *salk0333047* plants at 21 °C and after exposure to 30 °C. **b**) Comparisons of photosystem II activity (F_v_/F_m_) of high chlorophyll fluorescence (*hcf*) mutant with *salk0333047* plants at 21 °C and after exposure to 30 °C. (DOCX 40 kb)

## Abbreviations

Chl: Chlorophyll
ETR(I): PSI electron transport rate
ETR(II): PSII electron transport rate
F_0_: Minimum Chl fluorescence yield in the dark-adapted state
F_m_: Maximum Chl fluorescence yield in the dark-adapted state (Dark fluorescence yield)
F_m_′: Maximal fluorescence yield in the illuminated sample
F_0_/F_M_: A parameter related to changes in heat dissipation in the photosystem II antenna
FtsH: Filamentous temperature sensitive H
F_v_: Maximum Variable fluorescence defined as F_M_-F_0_
*F*_v_/F_m_: Maximum quantum yield of PSII photochemistry
HCF: High-chlorophyll-fluorescence
HL: High light
IC: Fluorescence induction curve
LC: Light response curve
LHC: Light harvesting complex
NPQ: Non-photochemical quenching
OEX: Oxygen-evolving complex
PAR: Photosynthetically active radiation
PSI: Photosystem I
PSII: Photosystem II
qN: Coefficient of nonphotochemical quenching
RC: Reaction center
ROS: Reactive oxygen species
SP: Saturating pulse (SP)
WT: Wild type
YII: Effective PSII quantum yield
