# Supplementary figures and images for: Chlorophyll fluorescence analysis revealed essential roles of FtsH11 protease in regulation of the adaptive responses of photosynthetic systems to high temperature
Source: BMC Plant Biol. 2018 Jan 10;18:11. doi: 10.1186/s12870-018-1228-2 (PMC5763919; doi:10.1186/s12870-018-1228-2)

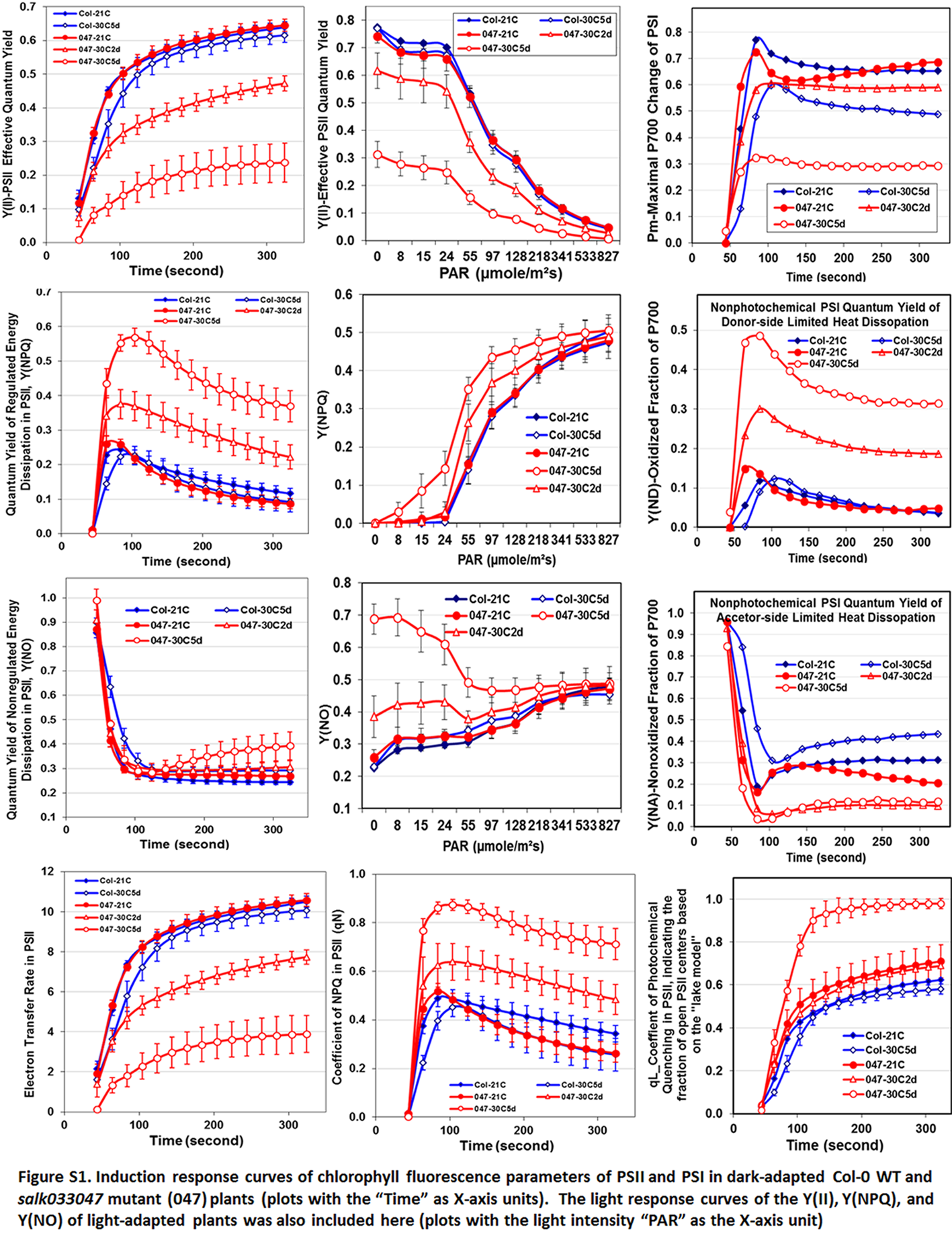

Supplement: Supplementary file 2 — Induction response curves of chlorophyll fluorescence parameters of PSII and PSI in dark adapted Col-0 WT and salk033047 mutant (047) plants (plots with the “Time” as X-axis units). The light response curves of the Y(II), Y(NPQ), and Y(NO) of light adapted plants was also included here (plots with the light intensity “PAR” as the X-axis unit). (TIFF 9702 kb) [file 12870_2018_1228_MOESM2_ESM.tif]

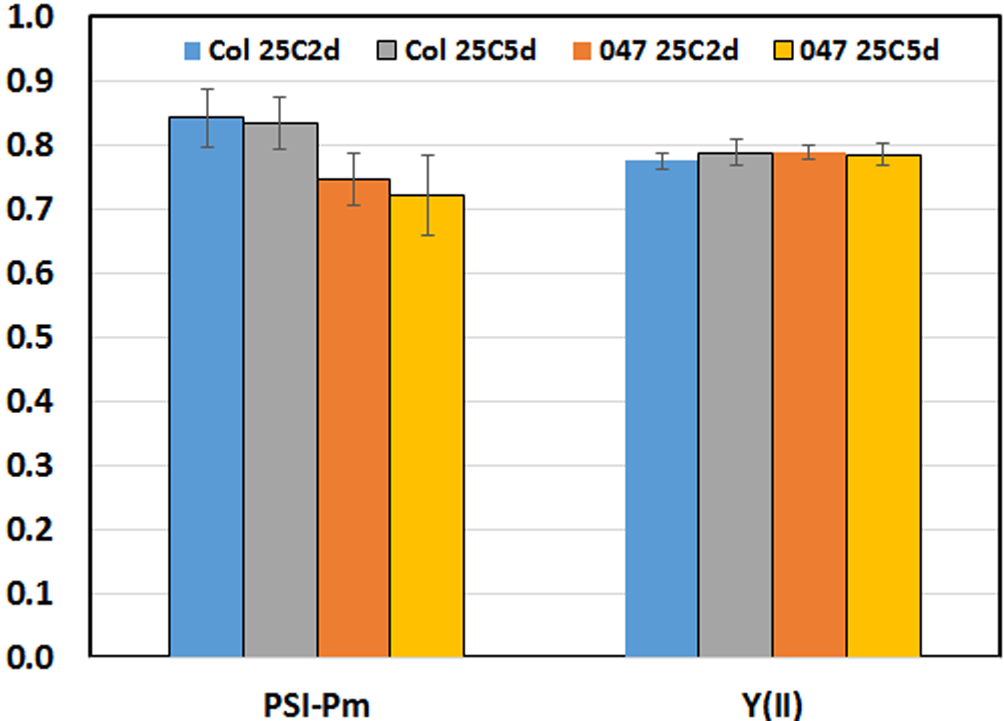

Supplement: Supplementary file 3 — Measured P700 of PSI (Pm) and photosynthetic efficiency of PSII (YII) changes in 2 and 5 day, 25 °C treated Col WT and FtsH11 mutant salk033047 (047) leaves. The 25 °C-treatet mutant plants showed marginal decreases only for Pm in dark adapted plants and PSI and PSII activities were determined immediately on DualPAM100. (TIFF 2259 kb) [file 12870_2018_1228_MOESM3_ESM.tif]

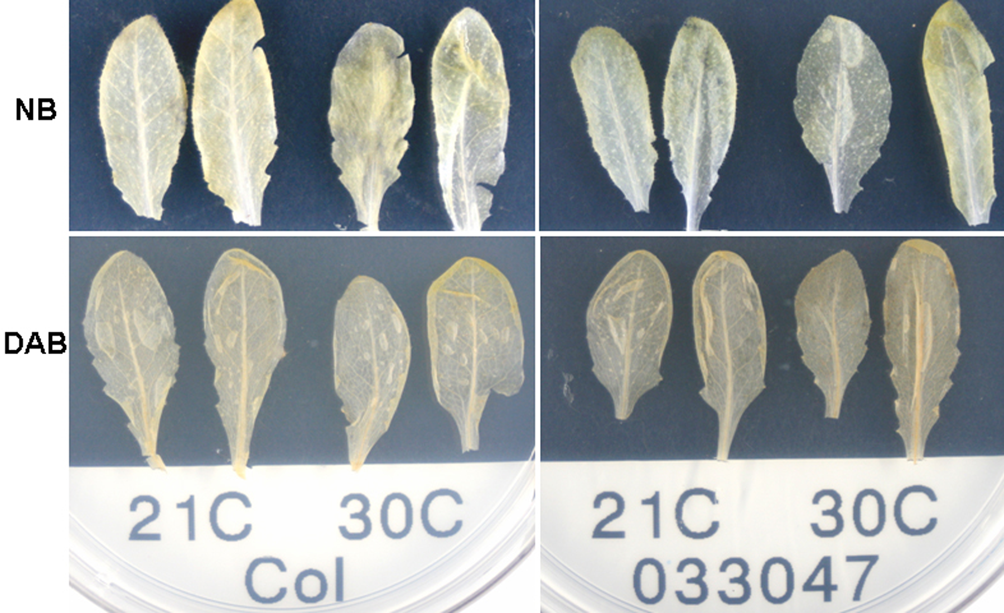

Supplement: Supplementary file 4 — Accumulation of ROS in rosette leaves of 21 °C-control and 30 °C-treated Col-0 WT and FtsH11 mutant salk033047 plants. The leave tissues were harvested and stained in nitroblue tetrazolium (NB) and 3,3′-diaminobenzidine (DAB) solution to examine the production of hydrogen peroxide and superoxide respectively. (TIFF 1831 kb) [file 12870_2018_1228_MOESM4_ESM.tif]
