# Supplementary material for: Chlorophyll fluorescence analysis revealed essential roles of FtsH11 protease in regulation of the adaptive responses of photosynthetic systems to high temperature
Source: BMC Plant Biol. 2018 Jan 10;18:11. doi: 10.1186/s12870-018-1228-2 (PMC5763919; doi:10.1186/s12870-018-1228-2)
Supplement: Supplementary file 5 — A) Comparisons of minimum chlorophyll fluorescence yield (Fo) of high chlorophyll fluorescence (hcf) mutant with salk0333047 plants at 21 °C and after exposure to 30 °C. b) Comparisons of photosystem II activity (Fv/Fm) of high chlorophyll fluorescence (hcf) mutant with salk0333047 plants at 21 °C and after exposure to 30 °C. (DOCX 40 kb) [file 12870_2018_1228_MOESM5_ESM.docx]

**Table S1a.** Comparisons of minimum chlorophyll fluorescence yield (F_o_) of high chlorophyll fluorescence (*hcf*) mutant with *salk0333047* plants at 21°C and after exposure to 30°C.

| **Mutant** | **Genotype** | **21**°**C** | **30**°**C 1d** | **30**°**C 2d** | **30**°**C 4d** | **30**°**C 7d** |
| --- | --- | --- | --- | --- | --- | --- |
| FtsH11 | SALK_033047 | 116.2 | 166.8 | 256.8 | 426.6 | 683.4 |
| HCF101 | CS319594 | 204.7 | 197.2 | 207.7 | 200.2 | 200.0 |
| HCF106 | CS834011 | 217.8 | 214.4 | 224.1 | 218.6 | 221.4 |
| HCF107 | SALK_062138 | 207.7 | 206.1 | 201.1 | 189.6 | 225.7 |
| HCF107 | SALK_079285C | 214.2 | 227.3 | 256.0 | 233.7 | 273.6 |
| HCF109 | SALK_094510 | 195.8 | 191.3 | 196.5 | 189.3 | 216.2 |
| HCF109 | SALK_144366 | 195.1 | 188.1 | 191.7 | 185.0 | 222.1 |
| HCF136 | CS803839 | 211.3 | 206.8 | 222.5 | 228.0 | 229.3 |
| HCF152 | SALK_010258 | 200.5 | 193.5 | 189.5 | 215.4 | 224.8 |
| HCF152 | SALK_010264 | 214.0 | 201.3 | 192.7 | 212.0 | 210.9 |
| HCF153 | CS27616 | 183.1 | 200.0 | 202.8 | 217.0 | 222.3 |
| HCF153 | CS831809 | 202.1 | 195.7 | 207.7 | 207.6 | 214.1 |
| HCF153 | SALK_015471C | 199.6 | 177.4 | 201.9 | 195.0 | 204.3 |
| HCF164 | SALK_147704 | 185.7 | 188.1 | 204.2 | 191.0 | 221.2 |
| HCF173 | SALK_035984 | 202.8 | 205.1 | 211.1 | 215.0 | 228.4 |
| HCF173 | SALK_004159 | 202.3 | 194.2 | 204.5 | 200.9 | 215.3 |
|  |  |  |  |  |  |  |

**Table S1b.** Comparisons of photosystem II activity (F_v_/F_m_) of high chlorophyll fluorescence (*hcf*) mutant with *salk0333047* plants at 21°C and after exposure to 30°C.

| **Mutant** | **Genotype** | **21**°**C** | **30**°**C 1d** | **30**°**C 2d** | **30**°**C 4d** | **30**°**C 7d** |
| --- | --- | --- | --- | --- | --- | --- |
|  |  | **F_v_/F_m_** | | | | |
| FtsH11 | SALK_033047 | 0.771 | 0.681 | 0.527 | 0.315 | 0.242 |
| HCF101 | CS319594 | 0.780 | 0.779 | 0.748 | 0.775 | 0.780 |
| HCF106 | CS834011 | 0.768 | 0.760 | 0.749 | 0.757 | 0.767 |
| HCF107 | SALK_062138 | 0.784 | 0.773 | 0.768 | 0.770 | 0.773 |
| HCF107 | SALK_079285C | 0.726 | 0.724 | 0.721 | 0.726 | 0.729 |
| HCF109 | SALK_094510 | 0.782 | 0.781 | 0.769 | 0.780 | 0.782 |
| HCF109 | SALK_144366 | 0.784 | 0.787 | 0.780 | 0.782 | 0.786 |
| HCF136 | CS803839 | 0.784 | 0.767 | 0.740 | 0.762 | 0.759 |
| HCF152 | SALK_010258 | 0.785 | 0.770 | 0.764 | 0.769 | 0.766 |
| HCF152 | SALK_010264 | 0.784 | 0.776 | 0.772 | 0.776 | 0.780 |
| HCF153 | CS27616 | 0.790 | 0.759 | 0.746 | 0.757 | 0.750 |
| HCF153 | CS831809 | 0.788 | 0.778 | 0.757 | 0.778 | 0.777 |
| HCF153 | SALK_015471C | 0.776 | 0.782 | 0.752 | 0.777 | 0.780 |
| HCF164 | SALK_147704 | 0.787 | 0.771 | 0.753 | 0.772 | 0.773 |
| HCF173 | SALK_035984 | 0.791 | 0.767 | 0.759 | 0.769 | 0.770 |
| HCF173 | SALK_004159 | 0.787 | 0.773 | 0.775 | 0.782 | 0.781 |
